# Cell fusion segregates progressive growth from metastasis.

**DOI:** 10.1038/bjc.1983.60

**Published:** 1983-03

**Authors:** E. Sidebottom, S. R. Clark

## Abstract

**Images:**


*Br. J. Cancer* (1983), **47**, 399–406

### Cell fusion segregates progressive growth from metastasis

E. Sidebottom & S.R. Clark

*Sir William Dunn School of Pathology, University of Oxford, Oxford OX1 3RE.*

**Summary** Cell fusion has been used to analyse the genetic determinants of metastasis at the cellular level. Highly metastatic mouse melanoma cells were fused with diploid mouse lymphocytes and a range of hybrid clones isolated and tested for tumorigenicity and metastatic potential by s.c. injection into newborn, histocompatible, sublethally-irradiated mice. Although almost all clones tested were tumorigenic, most had considerably reduced metastatic potential. This suggests that tumorigenicity and metastasis are determined by different genetic elements. Histological examination of primary tumours produced by metastatic and non-metastatic hybrid cell lines showed that an essential step in the production of metastases is the separation of tumour cells from the main tumour mass and their movement into the surrounding tissues. The primary tumours of a metastatic hybrid cell line showed local invasiveness whereas those of a non-metastatic cell line did not.

Although progressive growth is the property of tumour cells that has attracted most research effort, the properties that menace the life of a tumour-bearing host are invasiveness and the ability of the cells to establish distant metastases. The fact that, until recently, comparatively little effort had been devoted to the experimental investigation of invasion and metastasis reflects the difficulty of planning and carrying out informative experiments in this field. The situation is now changing, and experimental systems yielding interesting and important results have been introduced (Fidler, 1973; Nicolson & Winkelhake, 1975; Fidler & Kripke, 1977; Mareel *et al.*, 1979; Tao & Burger, 1977; Giavazzi *et al.*, 1980; Poste *et al.*, 1980).

One of the dilemmas in the investigation of metastasis is whether to treat the phenomenon as a single process or to break it down into its constituent parts and examine each separately. The overall process of metastasis is often divided into several stages. These usually include: 1) escape of cells from the primary tumour, 2) penetration of blood vessels, lymphatics or other channels of communication, 3) transport of cells via these routes, 4) arrest of circulating cells and their implantation on an appropriate surface, 5) repenetration of blood or lymph vessel walls and 6) growth of tumour cells at the new secondary site. Cells that fail to complete any one of these stages cannot form metastases.

In all the early experiments with the B16 melanoma cell lines the tumour cells were injected i.v. and metastatic potential was assessed by the formation of colonies in the lung. This method bypasses the first three steps of the overall process described above and measures a property that is now more usually referred to as "colonization potential." It has been shown that cells with a high colonization potential are not necessarily efficient at establishing spontaneous metastases after s.c. injection (Stackpole, 1981; Weiss *et al.*, 1982).

In the work described in the present paper all tumours were established by s.c. injection and metastatic potential was measured by observation of secondary deposits formed spontaneously in the lungs. All cells giving rise to metastases must therefore have passed through all the steps described above. It is clear that the isolation and characterization of tumour cell variants of different metastatic potential will be of importance in uncovering the mechanisms involved in metastasis (Poste & Fidler, 1980). We show here that cell fusion is a useful method for the generation of such variants.

### Materials and methods

### Cells

*Melanoma* All the melanoma cell lines were derived from a spontaneous tumour found in a C57B1 mouse by Parish and subsequently introduced by him into this laboratory (Jonasson *et al.*, 1977). Hypoxanthine-guanine phosphoribosyl transferase ($HPRT^-$) mutant cell lines were selected by growing wild type cells in 6-thioguanine ($5\,\mu g\,ml^{-1}$) after exposing them to ethyl methane sulphonate, ($200$ or $300\,\mu g\,ml^{-1}$) for 24 h. The thymidine kinase ($TK^-$) mutants were selected after growth for several generations in $100\,\mu g\,ml^{-1}$ bromodeoxyuridine followed by exposure to a bright white light source for 2 1/4 h. The derivation of the various cell lines, including the mutant cell lines, the "T" lines and the hybrid cell lines is shown in Figure 1. Cells were grown routinely in MEM.

Correspondence: E. Sidebottom.

Received 30 October 1982; accepted 12 December 1982

© The Macmillan Press Ltd., 1983

**Figure 1** Derivation of melanoma, mutant and hybrid cell lines.

medium (Gibco, Paisley, Scotland) supplemented with 10% new born calf serum (Gibco, or Sera-Lab., Crawley Down, Sussex). All cell lines were screened periodically for mycoplasma contamination, using Hoechst 33258 fluorochrome as described by Chen (1977) and found to be negative.

*Lymphocytes* The lymphocytes used in the cell fusions were obtained from teased lymph nodes excised from young adult CBA mice carrying the T6T6 translocation.

*Cell fusion*

This was done essentially as described by Harris & Watkins (1965) using UV-inactivated Sendai virus as the fusing agent and a 10:1 lymphocyte melanoma cell ratio; typically $10^7$ lymphocytes, $10^6$ melanoma cells. The hybrid clones were isolated in HAT medium (Littlefield, 1964).

*Assays of tumorigenicity & metastatic potential*

Unless otherwise stated all tests were made by injecting $5 \times 10^4$ cells, in 0.05 ml of phosphate-buffered saline, s.c. into the backs of newborn ($\leqslant 5$ day-old) sublethally-irradiated (4 Gy) syngeneic mice. For tests on hybrid cells the hosts were the F1 offspring of C57Bl/6 × CBA T6T6 matings. Since in principle the chances of generating metastases will increase with the duration of primary tumour growth, the primary tumours were allowed to grow for as long as was in practice possible. The animals were sacrificed when death from the tumour was impending. This end point has the advantage that it maximizes the probability of observing metastasis if the inoculum of cells is, in fact, capable of generating any.

Internal organs were examined for metastases visible to the naked eye. The lungs were removed, examined under a dissecting microscope and then fixed in formal saline and processed for routine histology. Other organs (liver, lymph nodes, kidney, spleen) were periodically examined under the dissecting microscope and by routine histology. In only a very small number of mice were metastases found in organs other than the lungs, and all the data recorded in the present paper refer to lung metastases. An animal was scored as positive for metastasis if one or more metastasis was identified unequivocally either by naked eye, under the dissecting microscope or in a histological section.

### Results

*Tumorigenicity & metastatic potential of melanoma cell lines*

Table I gives the tumour take incidence and the incidence of metastases of the parental tumour cells and the HPRT⁻ derivatives of them. The parental tumour cells gave 100% take incidences. Some of the HPRT⁻ derivatives fell short of this, but the overall take incidences remained very high. In the case of metastatic potential, however, selection for 6-thioguanine resistance produced a marked reduction. All the parental cells gave metastases in 70–80% of inoculated animals. For the HPRT⁻ derivatives this figure was from 0–40%. It thus appears that selection for thioguanine resistance also selects (probably co-incidentally) against metastatic potential. A cell line, Melgo-1, was derived from pooled metastatic nodules. This gave a 100% take incidence and a 81% incidence of metastases. When HPRT⁻ subclones of Melgo-1 were isolated and examined, although the take incidences remained 100%, the incidence of metastases varied from 0–77%. This type of variation in subclones has been described by Poste *et al.* (1981), who argue that heterogeneity of the cell population innoculated is required for the production of metastases. An equally plausible explanation is that the cells capable of generating metastases form only a small minority of the cell population injected so that individual subclones would be expected to show large variations in metastatic potential. One "T" line Melgo-1 6TG

**Table I** Tumorigenicity & metastasis of melanoma derivatives*

| Cell line | *Tumorigenicity** *no. 1° tumours* / *no. mice injected* | *Metastases* *no. with metastases* / *no. with 1° tumours* | % | Cell line | Tumorigenicity | Metastases | % |
|---|---|---|---|---|---|---|---|
| Parent cells | | | | | | | |
| MELJO | 24/24 | 19/24 | 79 | | | | |
| MEL 39-1 | 35/35 | 26/35 | 74 | | | | |
| MELRU | 21/21 | 15/21 | 71 | | | | |
| MELGO | 61/61 | 50/61 | 82 | Melgo T1 | 11/11 | 11/11 | 100 |
| MELGO-1 | 21/21 | 17/21 | 81 | | | | |
| HPRT⁻ Mutant cells | | | | | | | |
| PG19 | 29/30 | 6/29 | 21 | | | | |
| NuP2 | 16/16 | 3/16 | 19 | | | | |
| Cl 2C | 6/22 | 0/6 | 0 | | | | |
| Melgo-1 6TG | | | | | | | |
| Cl.2 | 6/6 | 0/6 | 0 | Cl.2 T1 | 11/11 | 3/11 | 27 |
| Cl.3 | 7/7 | 2/7 | 29 | | | | |
| Cl.4 | 13/13 | 5/13 | 39 | Cl.4 T1 | 7/7 | 2/7 | 29 |
| Cl.5 | 10/10 | 3/10 | 30 | Cl.5 T1 | 14/14 | 2/14 | 14 |
| Cl.6 | 8/8 | 1/8 | 13 | | | | |
| Cl.7 | 7/7 | 0/7 | 0 | | | | |
| Cl.8 | 11/11 | 3/11 | 27 | Cl.8 T1 | 35/35 | 27/35 | 77 |
| Cl.9 | 8/9 | 0/8 | 0 | | | | |

*All animals were sublethally irradiated syngeneic newborns and were injected with $5 \times 10^4$ cells s.c.

Cl.8T1, gave a 77% incidence of metastasis and maintained this high metastatic potential on further cultivation and subcloning. This "T" line was therefore used as the parent cell in the cell fusion experiments.

*Tumorigenicity & metastatic potential of hybrid cells*

*1. Hybrids between metastatic cells and diploid lymphocytes* Table II gives the incidence of primary tumours and metastases in a series of hybrid clones isolated from a cross between Melgo-1 6TG Cl.8T1 and diploid CBA T6T6 lymphocytes. These F.87 clones all gave 100% take incidence for primary tumours but gave a low incidence of metastases (0–17%) compared with the metastatic parent cell line (77%). Some of the hybrid clones and their tumours were examined karyologically by Dr. E.P. Evans (see Evans *et al.*, 1982 for Methods) and were shown to be triparental hybrids (2 melanoma × 1 lymphocyte) that had sustained substantial chromosome losses. It thus appears that the genetic determinants of progressive growth, as revealed by the incidence of primary tumours, may be dissociated, by cell fusion and subsequent chromosome segregation, from the determinants of metastasis.

When cells were isolated from primary tumours and retested no significant increase in metastatic potential was observed in most of the "T" lines, but one, F87 Cl 6T2, gave a high incidence of metastasis and has continued to do so on repeated cultivation and testing. These results support the view that cells capable of generating metastases are present in these cell populations in small numbers but enrichment for such cells can be achieved by selective procedures.

F87 Cl. 6T2 cells have a morphology that is easily distinguishable from the poorly metastatic lines (e.g. F87 Cl. 4T1). They are currently being used to study various biological and biochemical properties that have been postulated by other workers to be of importance in the production of metastases.

*2. Hybrids between two different tumour cell lines* Weiner *et al.* (1974) have shown that when different kinds of tumour cells are fused together, the resulting hybrids are usually tumorigenic. It was therefore of interest to examine the consequences of fusing together two metastatic cell lines. We have not been able to isolate a thymidine kinase⁻ variant of the melanoma with a metastatic potential as high as that of Melgo 1 6TG Cl. 8T1, but

**Table II** Tumorigenicity & metastasis of hybrid clones (Melgo-1 6TG Cl.8 T1 × CBA $T_6T_6$ lymphocytes) F.87

| Cell line | *Tumorigenicity* *no. 1° tumours* / *no. mice injected* | *Metastases* *no. with metastases* / *no. with 1° tumours* | *Cell line* | *Tumorigenicity* | *Metastases* |
|---|---|---|---|---|---|
| Melanoma | | | | | |
| Parent | 35/35 | 27/35 | | | |
| Hybrid F87 Cl.1 | 9/9 | 0/9 | Cl.1 T1 | 13/13 | 0/13 |
| F87 Cl.3 | 11/11 | 0/11 | Cl.3 T1 | 16/16 | 1/16 |
| F87 Cl.4 | 8/8 | 0/8 | Cl.4 T1 | 37/37 | 0/37 |
| F87 Cl.5 | 12/12 | 0/12 | Cl.5 T2 | 9/9 | 2/9 |
| F87 Cl.6 | 12/12 | 2/12 | Cl.6 T1 | 18/18 | 10/18 |
| | | | Cl.6 T2 | 77/77 | 73/77 |
| F87 Cl.7 | 56/56 | 1/56 | Cl.7 T1 | 14/14 | 4/14 |
| F87 Cl.8 | 15/15 | 2/15 | Cl.8 T1 | 8/8 | 0/8 |

Melgo-1 $TK^-$ Cl. 7 gave an incidence of metastases of between 20 and 30%, and therefore provided an adequate, if not ideal, parent cell. The results of tests on F92 hybrid clones derived from the fusion of Melgo-1 6TG Cl. 8T1 with Melgo-1 $TK^-$ Cl. 7 are shown in Table III. In agreement with Weiner *et al.* (1974), we found that all the hybrid clones gave a 100% take incidence, like the parent cells. However, the incidence of metastases was greatly reduced in the hybrid clones, the majority producing no metastases at all. This low metastatic potential was maintained on cells isolated directly from the primary tumours (the T1 series). These results provide further evidence that the genetic determinants of progressive growth can be segregated by cell fusion from the genetic determinants of metastasis.

*Histological findings*

We have made a systematic histological examination of the tumours produced by clones that generate metastases and those that do not. The observations will be reported in detail elsewhere, but the essential features are as follows. The social behaviour of cells in the primary tumours produced by metastatic cell lines can be easily distinguished from that of the cells in the primary tumours produced by non-metastatic cell lines. In tumours produced by cell lines that give rise to metastases, the cells at the periphery of the tumour move away from the main tumour mass and migrate into the surrounding tissues (Figure 2). In tumours produced by cell lines that do not give rise to metastases, the cells at the periphery of the tumour remain attached

**Table III** Tumorigenicity & metastasis of hybrid clones between two metastatic cell lines (Melgo-1 6TG Cl.8 T1 × Melgo-1 $TK^-$ Cl.7) F92

| Cell line | *Tumorigenicity* *no. 1° tumours* / *no. mice* | *Metastases* *no. with metastases* / *no. with 1° tumours* | *Cell line* | *Tumorigenicity* | *Metastases* |
|---|---|---|---|---|---|
| Melgo-1 | | | | | |
| 6TG Cl.8 T1 | 35/35 | 27/35 | | | |
| Melgo-1 | | | | | |
| $TK^-$Cl 7 | 19/19 | 5/19 | | | |
| Hybrid F92 Cl.1 | 11/12 | 1/11 | Cl.1 T1 | 13/13 | 0/13 |
| F92 Cl.2 | 17/17 | 1/17 | Cl.2 T1 | 14/14 | 1/14 |
| F92 Cl.3 | 14/14 | 2/14 | Cl.3 T1 | 9/9 | 0/9 |
| | | | Cl.3 T2 | 12/12 | 0/12 |
| F92 Cl.4 | 11/14 | 0/11 | Cl.4 T1 | 10/11 | 0/10 |
| F92 Cl.5 | 11/11 | 0/11 | Cl.5 T1 | 14/14 | 0/14 |
| F92 Cl.6 | 9/9 | 1/9 | Cl.6 T1 | 15/15 | 0/15 |
| F92 Cl.7 | 11/11 | 0/11 | Cl.7 T1 | 18/18 | 0/18 |
| F92 Cl.8 | 13/13 | 0/13 | Cl.8 T1 | 13/13 | 0/13 |
| F92 Cl.9 | 16/16 | 0/16 | | | |
| F92 Cl.10 | 16/17 | 0/16 | | | |

**Figure 2** Periphery of primary tumours growing in the s.c. tissues of newborn mice after the injection of $5 \times 10^4$ F87Cl6T2 (metastatic cells). (a) 10d after injection. The edge of the tumour is ill-defined and clumps of tumour cells have separated from the main tumour mass (arrows). (b) 7d. Tumour cells are invading through muscle into the dermis (arrows).

to the main tumour mass (Figure 3) and apparently do not become disseminated even when the tumour has grown into the lumen of a vessel (Figure 4). These findings indicate that an essential element in the production of spontaneous metastases from a solid tumour growing in an s.c. site is the extent to which the cells in the tumour cohere or fail to cohere. This property must be determined by genetic elements that are different from those that determine the progressive growth of the primary tumour itself.

### Discussion

Two simple conclusions can be drawn from the experiments described here. First it is possible to make hybrids between malignant and normal cells in which different hybrid clones vary considerably in their ability to produce metastases. Second, in these segregant clones, tumorigenicity and metastatic potential are easily dissociated. On the simplest hypothesis that both tumorgenicity and metastatic potential are genetically determined the genes determining the one cannot be closely linked to those determining the other.

The experiments show further that cell fusion can be used to analyse metastasis in much the same way as has been used to analyse progressive growth (Harris *et al.*, 1969; Harris, 1971, 1975). Since metastatic potential segregates in hybrid cells, matched pairs of metastatic and non-metastatic hybrids can be used to search for biological or biochemical markers that are linked to the ability of tumour cells to produce secondary deposits. We have begun to use such matched pairs of hybrids as screens for markers linked to metastasis and the histological difference reported here in the behaviour of primary tumours derived from metastatic and non-metastatic hybrid cell lines is an example of a marker detected in this way.

**Figure 3** Periphery of primary tumours growing in the s.c. tissues of newborn mice 14 d after injection $5 \times 10^4$ F87C14T1 (non-metastatic) cells. In both tumours the edge is clearly defined (arrows), no invasion is seen (a) Pigmented tumour (b) unpigmented tumour.

**Figure 4** Clump of F87Cl4T1 (non-metastatic) cells within the lumen of a blood vessel in the subcutaneous tissues of a newborn mouse, 24 h after injection.

The authors thank Professor H. Harris for his encouragement and advice during the experimental work and the preparation of the manuscript, and Mr. D.F. Etheridge for the histology. The work was supported by the Cancer Research Campaign.

### References

CHEN, T.R. (1977). *In situ* detection of mycoplasma contamination in cell cultures by fluorescent Hoeschst 33258 Stain. *Exp. Cell Res.*, **104**, 255.

EVANS, E.P., BURTENSHAW, M.D., BROWN, B.B., HENNION, R. & HARRIS, H. (1982). The analysis of malignancy by cell fusion IX. Re-examination and clarification of the cytogenetic problem. *J. Cell Sci.*, **56**, 113.

FIDLER, I.J. (1973). Selection of successive tumour lines for metastasis. *Nature*, (New Biol.) **242**, 148.

FIDLER, I.J. & KRIPKE, M.L. (1977). Metastasis results from pre-existing variant cells within a malignant tumour. *Science*, **197**, 893.

GIAVAZZI, R., ALESSANDRI, G., SPREAFICO, F., GARATTINI, S. & MANTOVANI, A. (1980). Metastasizing capacity of tumour cells from spontaneous metastases of transplanted murine tumours. *Br. J. Cancer*, **42**, 462.

HARRIS, H. (1971). Cell fusion and the analysis of malignancy. The Croonian lecture. *Proc. R. Soc. B.*, **179**, 1.

HARRIS, H. (1975). Cell fusion and the analysis of malignancy. London: *Cancer Research Campaign*. (53rd Annual Report).

HARRIS, H., MILLER, O.J., KLEIN, G., WORST, P. & TACHIBANA, T. (1969). Suppression of malignancy by cell fusion. *Nature*, **233**, 363.

HARRIS, H. & WATKINS, J.F. (1965). Hybrid cells derived from mouse and man: Artificial heterokaryons of mammalian cells from different species. *Nature*, **205**, 640.

JONASSON, V., POVEY, S. & HARRIS, H. (1977). The analysis of malignancy by cell fusion. VII Cytogenetic analysis of hybrids between malignant and diploid cells and of tumours derived from them. *J. Cell Sci.*, **24**, 217.

LITTLEFIELD, J.W. (1964). Selection of hybrids from matings of fibroblasts *in vitro* and their presumed recombinants. *Science*, **145**, 709.

MAREEL, M., KINT, J. & MAYVISCH, C. (1979). Methods of study of the invasion of malignant C3H-mouse fibroblasts into chick heart *in vitro*. *Virchows Arch. B. Cell Pathol.*, **30**, 95.

NICHOLSON, G.L. & WINKELHAKE, J.L. (1975). Organ specificity of blood-borne tumour metastasis deformed by cell adhesion. *Nature*, **255**, 230.

POSTE, G., DOLL, J. & FIDLER, I.J. (1981). Interactions among clonal subpopulations affect stability of the metastatic phenotype in polyclonal populations of B16 melanoma cells. *Proc. Natl Acad. Sci.*, **78**, 6226.

POSTE, G., DOLL, J., HART, I.R. & FIDLER, I.J. (1980). *In vitro* selection of murine B16 melanoma variants with enhanced tissue-invasive properties. *Cancer Res.*, **40**, 1636.

POSTE, G. & FIDLER, I.J. (1980). The pathogenesis of cancer metastasis. *Nature*, **283**, 139.

STACKPOLE, C.W. (1981). Distinct lung colonizing and lung metastasizing cell populations in B16 mouse melanoma. *Nature*, **289**, 798.

TAO, T.W. & BURGER, M.M. (1977). Non-metastasizing variants selected from metastasizing melanoma cells. *Nature*, **270**, 437.

WEISS, L., MAYHEW, E., RAPP, D.G. & HOLMES, V.C. (1982). Metastatic inefficiency in mice bearing B16 melanomas. *Br. J. Cancer*, **45**, 44.

WIENER, F., KLEIN, G. & HARRIS, H. (1974). The analysis of malignancy by cell fusion VI. Hybrids between different tumour cells. *J. Cell Sci.*, **16**, 189.


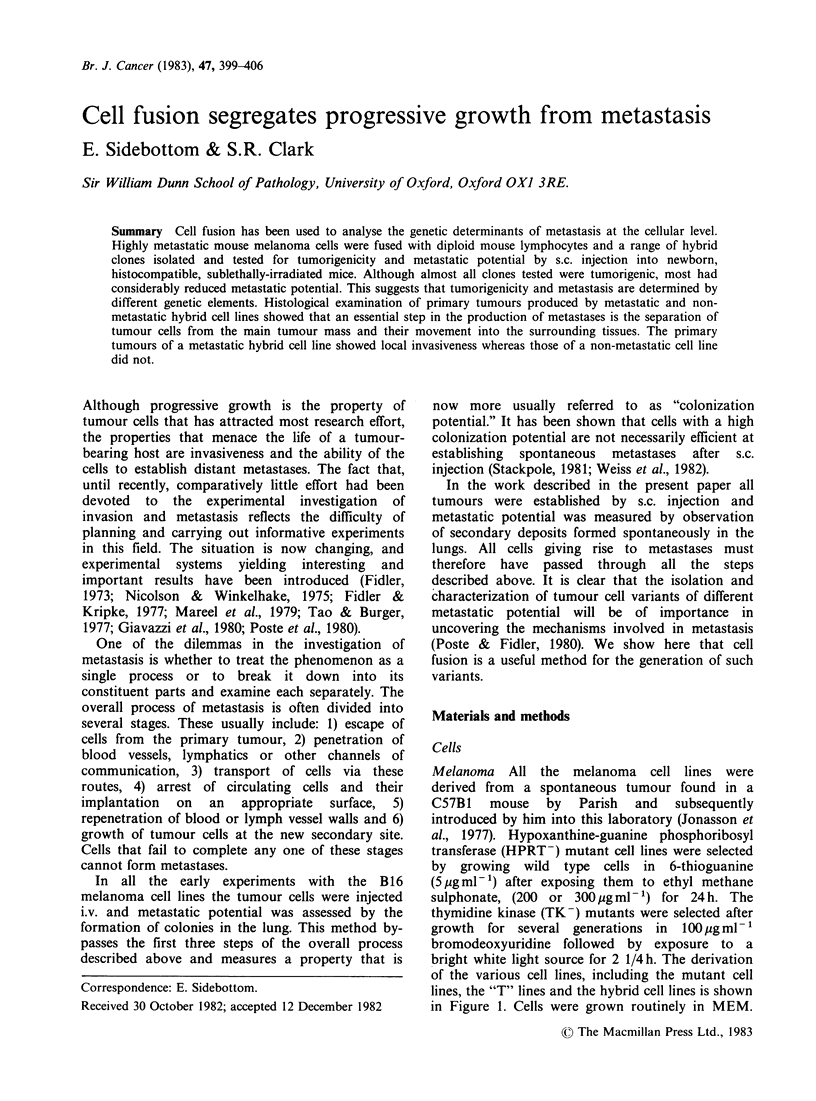

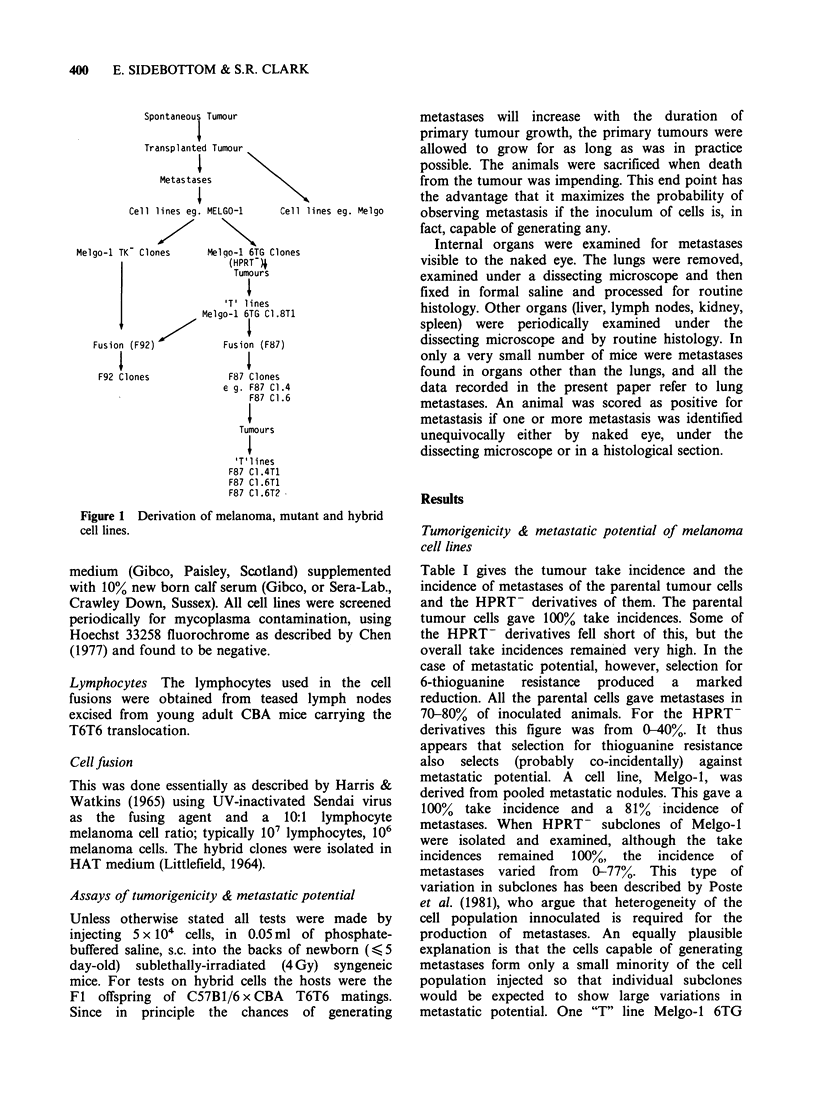

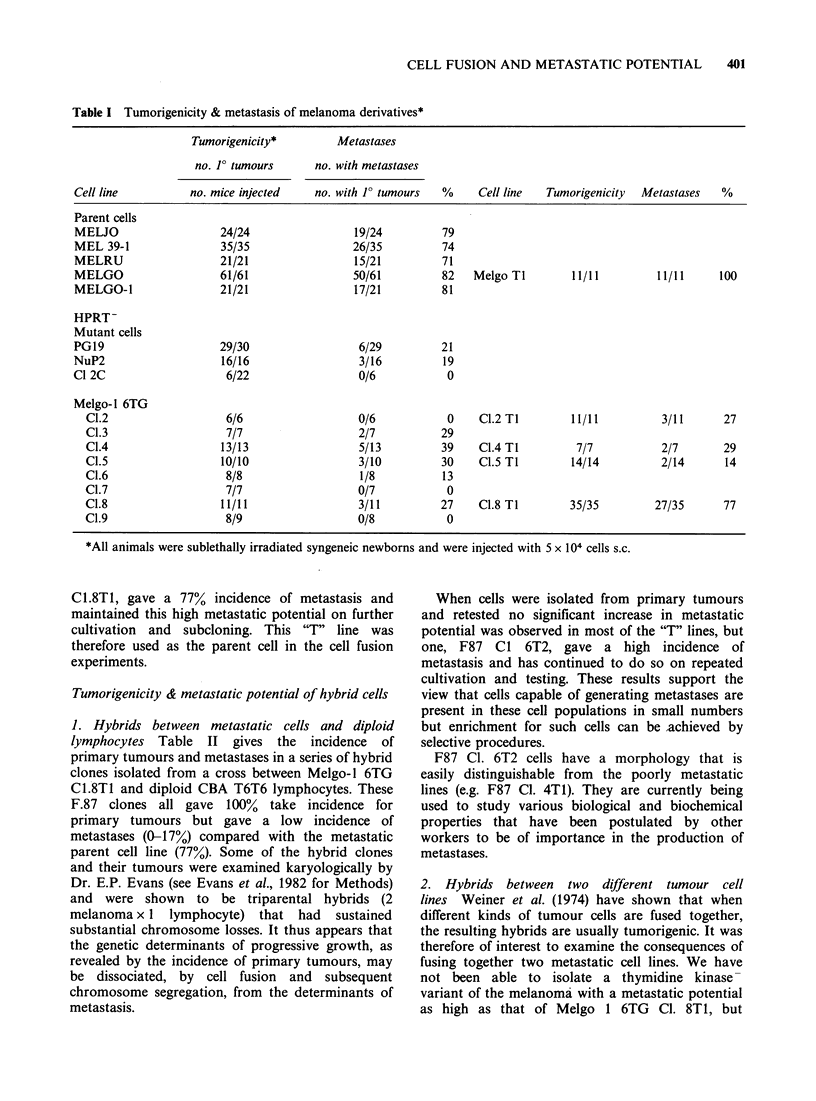

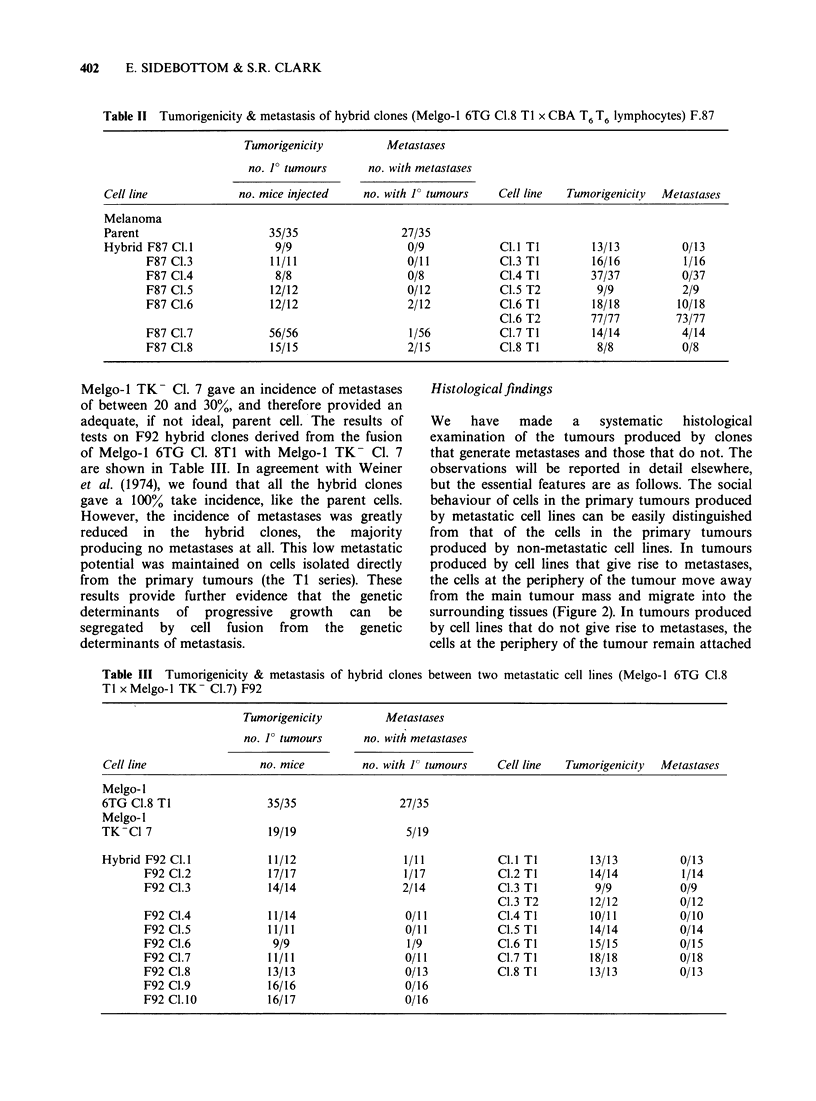

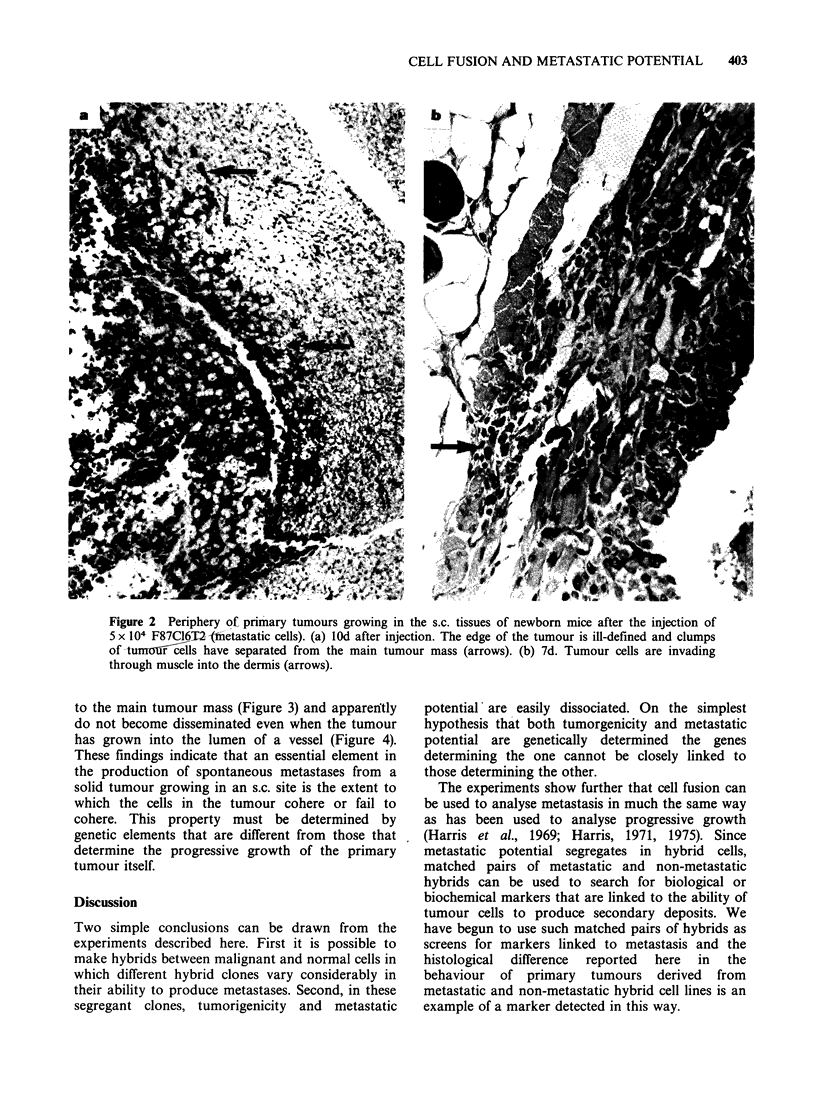

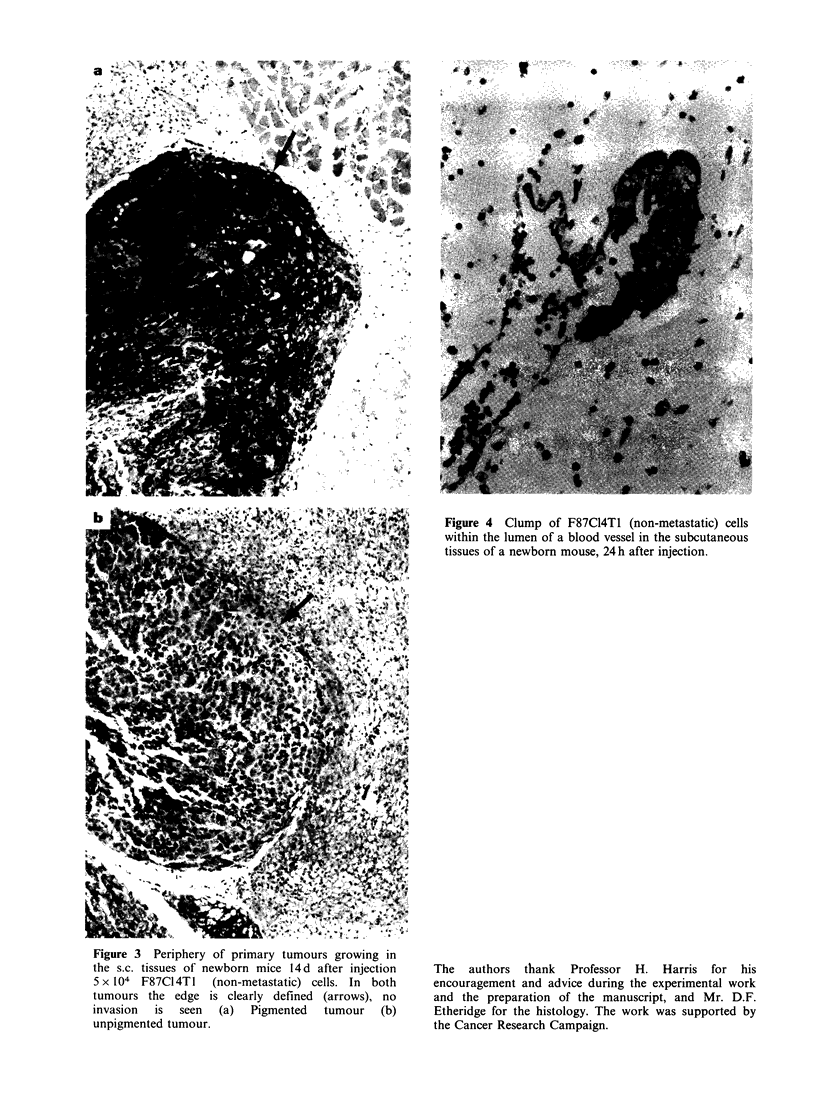

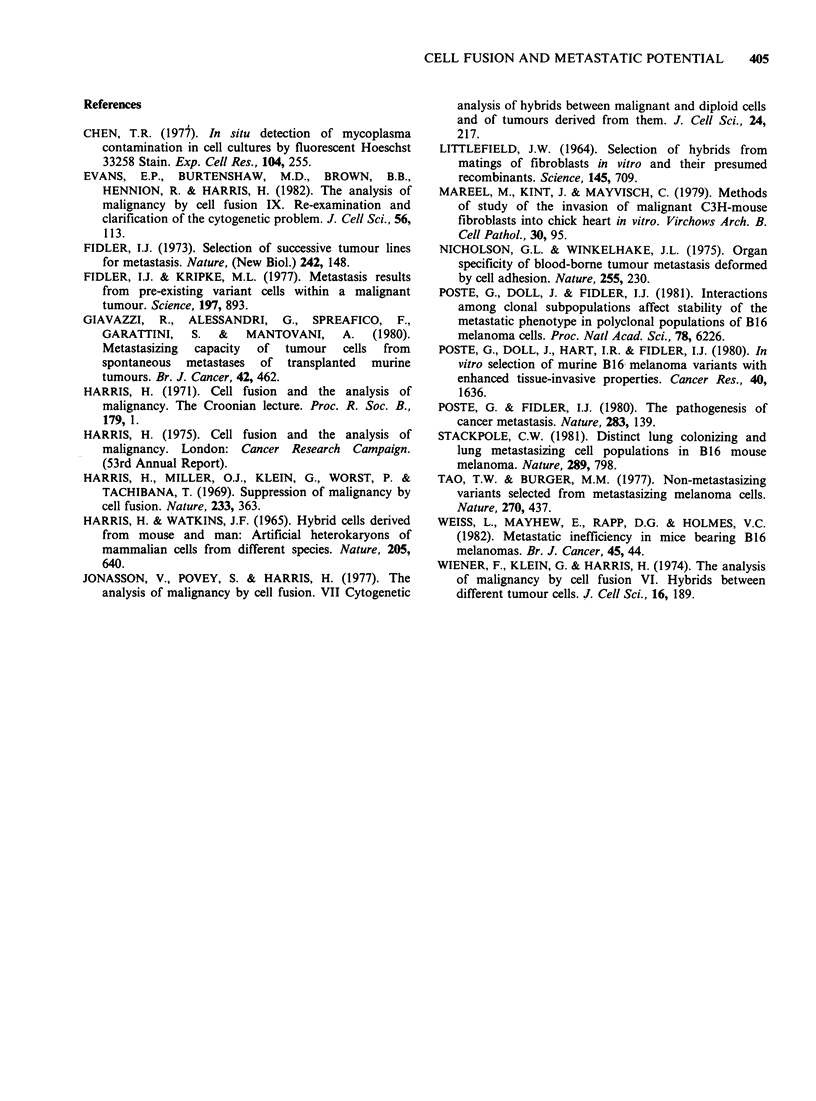


## References

[OCR_00511] Chen T. R. (1977). In situ detection of mycoplasma contamination in cell cultures by fluorescent Hoechst 33258 stain.. Exp Cell Res.

[OCR_00516] Evans E. P., Burtenshaw M. D., Brown B. B., Hennion R., Harris H. (1982). The analysis of malignancy by cell fusion. IX. Re-examination and clarification of the cytogenetic problem.. J Cell Sci.

[OCR_00527] Fidler I. J., Kripke M. L. (1977). Metastasis results from preexisting variant cells within a malignant tumor.. Science.

[OCR_00523] Fidler I. J. (1973). Selection of successive tumour lines for metastasis.. Nat New Biol.

[OCR_00532] Giavazzi R., Alessandri G., Spreafico F., Garattini S., Mantovani A. (1980). Metastasizing capacity of tumour cells from spontaneous metastases of transplanted murine tumours.. Br J Cancer.

[OCR_00554] HARRIS H., WATKINS J. F. (1965). HYBRID CELLS DERIVED FROM MOUSE AND MAN: ARTIFICIAL HETEROKARYONS OF MAMMALIAN CELLS FROM DIFFERENT SPECIES.. Nature.

[OCR_00539] Harris H. (1971). Cell fusion and the analysis of malignancy.. Proc R Soc Lond B Biol Sci.

[OCR_00549] Harris H., Miller O. J., Klein G., Worst P., Tachibana T. (1969). Suppression of malignancy by cell fusion.. Nature.

[OCR_00560] Jonasson J., Povey S., Harris H. (1977). The analysis of malignancy by cell fusion. VII. Cytogenetic analysis of hybrids between malignant and diploid cells and of tumours derived from them.. J Cell Sci.

[OCR_00568] LITTLEFIELD J. W. (1964). SELECTION OF HYBRIDS FROM MATINGS OF FIBROBLASTS IN VITRO AND THEIR PRESUMED RECOMBINANTS.. Science.

[OCR_00573] Mareel M., Kint J., Meyvisch C. (1979). Methods of study of the invasion of malignant C3H-mouse fibroblasts into embryonic chick heart in vitro.. Virchows Arch B Cell Pathol Incl Mol Pathol.

[OCR_00579] Nicolson G. L., Winkelhake J. L. (1975). Organ specificity of blood-borne tumour metastasis determined by cell adhesion?. Nature.

[OCR_00584] Poste G., Doll J., Fidler I. J. (1981). Interactions among clonal subpopulations affect stability of the metastatic phenotype in polyclonal populations of B16 melanoma cells.. Proc Natl Acad Sci U S A.

[OCR_00590] Poste G., Doll J., Hart I. R., Fidler I. J. (1980). In vitro selection of murine B16 melanoma variants with enhanced tissue-invasive properties.. Cancer Res.

[OCR_00596] Poste G., Fidler I. J. (1980). The pathogenesis of cancer metastasis.. Nature.

[OCR_00600] Stackpole C. W. (1981). Distinct lung-colonizing and lung-metastasizing cell populations in B16 mouse melanoma.. Nature.

[OCR_00605] Tao T. W., Burger M. M. (1977). Non-metastasising variants selected from metastasising melanoma cells.. Nature.

[OCR_00610] Weiss L., Mayhew E., Rapp D. G., Holmes J. C. (1982). Metastatic inefficiency in mice bearing B16 melanomas.. Br J Cancer.

[OCR_00615] Wiener F., Klein G., Harris H. (1974). The analysis of malignancy by cell fusion. VI. Hybrids between different tumour cells.. J Cell Sci.

